# MRI and Ultrasound Visualization of a Nerve Repair Implant Containing Nitinol

**DOI:** 10.1097/GOX.0000000000006063

**Published:** 2024-08-09

**Authors:** Michelle Akerman, Darryl B. Sneag, Lisa Gfrerer, Yoshimi Endo, Alyssa B. Valenti, Isaac P. Clements, Ek T. Tan

**Affiliations:** From the * Radiology and Imaging, Hospital for Special Surgery, New York, N.Y.; † Plastic and Reconstructive Surgery, Weill Cornell Medicine, New York, N.Y.; ‡ BioCircuit Technologies, Inc., Atlanta, Ga.

## Abstract

**Background:**

Nerve Tape is a novel nerve repair device containing nitinol microhooks that provide sutureless attachment for nerve coaptation. This study evaluated visualization of Nerve Tape on magnetic resonance imaging (MRI) and ultrasound, with the objective of exploring its potential as an imaging marker for localizing nerve repair sites.

**Methods:**

Phantom imaging experiments were first conducted to assess the visibility of Nerve Tape on MRI and ultrasound. A cadaveric limb investigation was then performed to further characterize the magnetic susceptibility patterns of Nerve Tape and to confirm its localization at the repair site.

**Results:**

Phantom imaging experiments demonstrated clear visualization of Nerve Tape on both MRI and ultrasound, with Nerve Tape microhooks appearing as signal voids on MRI and hyperechoic foci on ultrasound. Subsequent cadaveric limb investigation further characterized Nerve Tape’s magnetic susceptibility patterns and confirmed localization of the device at the repair site. The physical dimensions of Nerve Tape and locations observed on both MRI and ultrasound matched design and measurements made during surgery. Measurement discrepancies could be attributed to magnetic susceptibility artifacts in MRI, and to comet tail and shadowing effects in ultrasound.

**Conclusions:**

Repairs performed with Nerve Tape can be reliably localized for imaging, potentially facilitating assessment of repair site integrity and further advancement toward image-based monitoring of nerve regeneration. Further research, including in vivo human studies, is warranted to confirm these preliminary findings.

**Takeaways**

**Question:** Can a nerve repair implant provide magnetic resonance imaging (MRI) and ultrasound-based localization of the peripheral nerve repair site?

**Findings:** Phantom and cadaveric limb investigations demonstrate reliable visualization and localization of the nerve repair implant on MRI and ultrasound. The physical dimensions of nerve repair implants match design specifications, and spatial locations match experiment designs.

**Meaning:** Noninvasive localization of the nerve repair site aids peripheral nerve assessment using either MRI or ultrasound, including potentially facilitating assessment of the repair site integrity and detection of nerve regeneration.

## INTRODUCTION

Traumatic peripheral nerve injuries are prevalent with an annual incidence of 16.9 per 100,000 persons in the United States.^1^ Most commonly due to lacerations, traumatic nerve injuries can lead to permanent sensorimotor deficits, including chronic neuropathic pain. Although peripheral nerves carry inherent capacity for spontaneous regeneration, this natural repair process is limited, and surgical intervention is often required to physically reattach, or coapt, severed nerve ends.^2^ Surgical nerve repairs are also frequently performed for the reinnervation of breast mastectomy skin flaps after ablative surgery, restoration of facial nerve function in the context of congenital anomalies or oncologic resections, postamputation pain control, prosthesis management, and migraine treatment.

The current standard for direct nerve repair is microsuture neurorrhaphy, a technique requiring advanced technical skills and with varying repair efficacy, ranging from 24% to 81%,^3^ in part due to difficulties in nerve approximation.^4^ Proper fascicular alignment, nerve coaptation without tension, and nerve stump debridement are crucial factors that impact outcomes.

Nerve Tape (NT; BioCircuit Technologies, Inc., Atlanta, Ga.) was developed as an alternative to traditional suture techniques for nerve coaptation. The device contains microscale hooks made of nitinol, a nickel-titanium alloy with superelastic properties commonly used in medical devices (including cardiac stents). These microhooks, designed to grip and stabilize tissue, are embedded within a thin, flexible backing layer of decellularized porcine small intestinal submucosa (SIS).^4^ (**See figure, Supplemental Digital Content 1**, which displays NT structure and application in nerve repair. A, NT contains columns of nitinol microhooks integrated within a biologic backing layer. B, Severed nerve ends are placed onto each side of the device, where opposing banks of microhooks engage the epineurium. The device is then wrapped around the approximated nerve ends to facilitate additional microhook engagement and achieve closure. http://links.lww.com/PRSGO/D427.)

The microhooks penetrate the outer epineurium of the nerve, whereas the SIS extracellular matrix backing envelops the coaptation site to provide a stable, entubulated repair. NT circumvents some of the technical complexities associated with microsurgical techniques. The microhooks are arranged to distribute and disperse tension away from the nerve coaptation site, which has been shown in experimental models to enhance axon regeneration.^5,6^ Prior cadaver-based testing of the NT compared with microsuture repairs demonstrated significantly reduced surgical time, enhanced repair strength, and improved nerve end alignment (even when repairs were performed by less-experienced surgeons).^7^ In preclinical testing, NT has been shown to provide superior nerve conduction recovery and comparable axon regeneration efficacy when compared with microsuture or conduit-assisted repairs.^4^

Despite recent advancements in magnetic resonance imaging (MRI) and ultrasound technology, postoperative imaging of the nerve repair site is infrequently obtained in routine clinical practice. Although diagnostic imaging cost and complexity are undoubtedly contributing factors, another challenge arises from the difficulty in identifying the suture material that delineates the neurorrhaphy repair site. The resulting uncertainty in repair site localization limits the assessment of repair site integrity and early identification of potential issues that can hinder nerve regeneration, such as dehiscence or neuroma formation.

Suture material typically used for end-to-end direct nerve repair ranges from 8-0 to 11-0 monofilament nylon.^8^ This material, known for its insensitivity to magnetic forces, induces minimal MR image artifacts and no distortion.^9^ The limited MRI visibility of this suture is attributed to nylon’s MR relaxivity properties and the suture’s fine diameters (0.030–0.039 mm).^10^ In two-dimensional (2D) MRI, in-plane resolution acquired is typically within the range of 0.4–0.5 mm,^11^ well surpassing the suture’s diameter. In ultrasonography, nylon sutures appear as linear hyperechoic foci,^12^ aiding in their identification, but the high spatial resolution of up to 0.05–0.1 mm of modern ultrasound probes is depth-dependent and may not suffice in identifying the suture material.

This work explores whether NT’s metallic microstructures might provide a secondary advantage of facilitating imaging-based localization and assessment of nerve repairs. We hypothesized that microhooks would exhibit magnetic susceptibility and cause signal voids, rendering them easily identifiable on MRI, and would also be discernible on high-resolution ultrasound for definitive localization of the repair site. The ability to easily identify the coaptation site could prove valuable for early assessment of nerve repair viability and for monitoring and understanding the mechanisms of successful nerve regeneration through noninvasive imaging. As magnetic susceptibility on MRI and comet tail and shadowing on ultrasound can also be a significant source of image artifact and obscure adjacent tissue, we also aimed to characterize the extent of artifact caused by this device on both MRI and high-resolution ultrasound, two commonly used imaging modalities for evaluating peripheral nerve injuries.

## MATERIALS AND METHODS

### MRI Phantom Preparation

The visibility of NT was assessed in an agarose-based phantom. The phantom design consisted of nerve-mimicking cylindrical components, wrapped by NT devices, and these were then embedded in a muscle-mimicking component. These components were all contained in a 7-oz (207-mL) glass jar with a glass lid.

To prepare nerve-mimicking components at 3.0 Tesla (T) field strength, the target longitudinal relaxation (T1) and transverse relaxation (T2) times for peripheral nerves were 1400 and 60–80 ms, respectively.^13^ Agarose doped with gadolinium was utilized, with the concentrations of agarose and gadolinium needed to achieve these target T1 and T2 values.^14^ The nerve-mimicking samples were prepared in a glass beaker by combining a 0.05% NaN_3_ solution (Sigma-Aldrich; Milwaukee, Wis.), 2% agarose (Sigma-Aldrich), and 50 µmol/kg of gadobutrol (Gadovist, Bayer AG, Leverkusen, Germany). The compound was mixed at room temperature continuously and heated to 75°C, with deionized water added to maintain the original mixture mass to account for evaporation. Once the agarose was completely dissolved and the mixture appeared uniform, it was poured into 50-mL polypropylene tubes and allowed to cool overnight to room temperature. Three cylindrical shapes with nominal diameters of 9, 5, and 7 mm, representing nerves with varying diameters, were formed by separately pushing 2- and 5-mL serological pipettes and a 9-mm internal-diameter straw through the agarose, before extracting the cylinders. Each cylinder was placed onto three separate NT devices with the microhooks side up, similar to the manufacturer-recommended implantation procedure; a slight downward pressure was applied to each cylinder to engage the hooks before wrapping NT around them **(Supplemental Digital Content 1**, http://links.lww.com/PRSGO/D427). The diameters of the nerve-mimicking cylinders (9, 5, and 7 mm) were chosen to approximate the intended diameters of the implanted NT devices (7, 4, and 7 mm for NT_1_, NT_2_, and NT_3_, respectively).

**Fig. 1.**
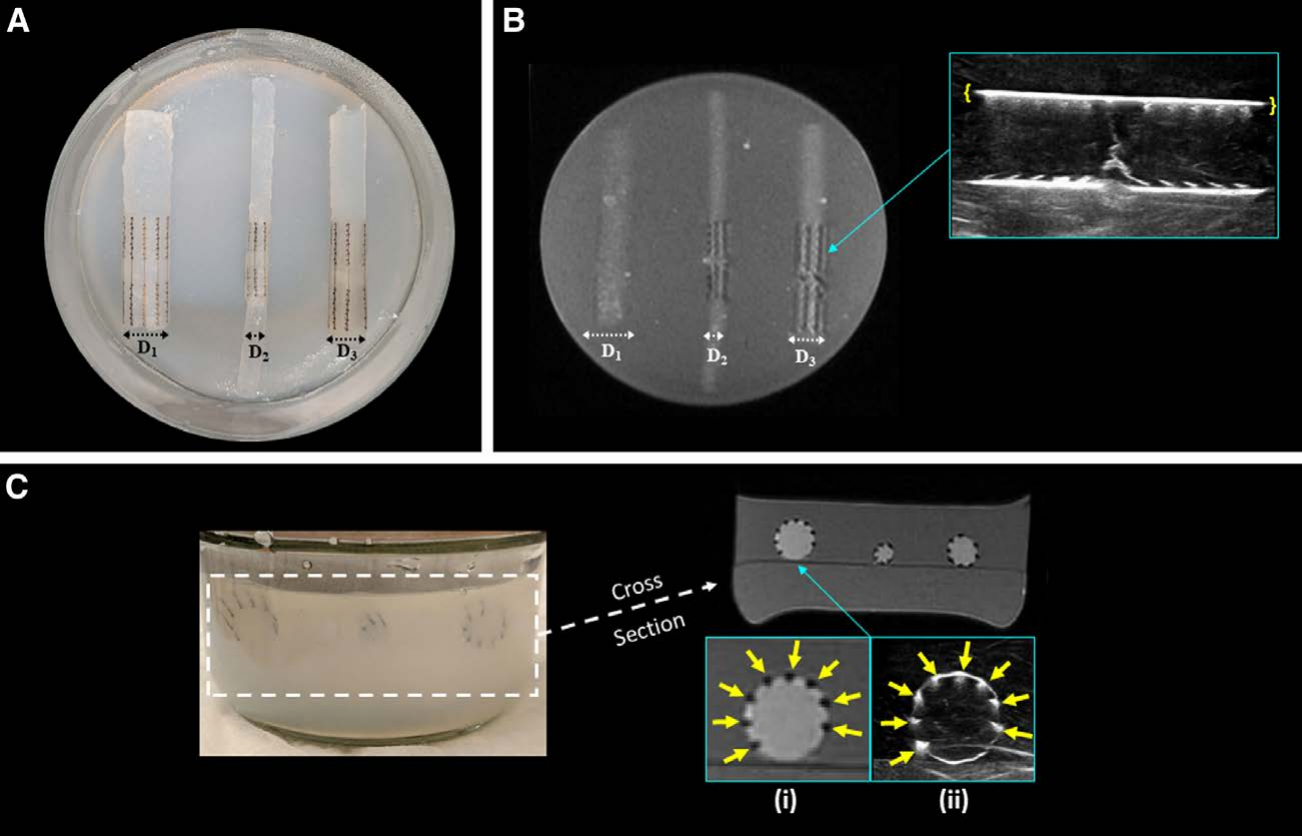
Visualization of nerve-mimicking components with NT: MRI and ultrasound imaging. A, Top and (C) front views of the agarose phantom with three nerve-mimicking cylinder components of varying diameters (D_1_ = 9 mm, D_2_ = 5 mm, D_3_ = 7 mm) each wrapped in NT devices and embedded in parallel within a muscle component. The nerve and muscle components had different targeted T2 values (70 and 35 ms, respectively) to reflect higher signal in the nerve components. B, Coronal MR image, capturing the slice longitudinal to two of the nerve components, shows signal voids corresponding to the nitinol columns and microhooks of the NT devices (cylinders D_2_ and D_3_, slice with cylinder D_1_ outside of image plane). Magnified inset displays longitudinal ultrasound image of NT_3_ (yellow brackets at device ends). C i, MRI slice acquired axial to the nerve cylinders with the magnified inset demonstrating microhooks with focal signal voids (yellow arrows). C ii, Ultrasound image featuring signal hyperintensity (yellow arrows) in the locations of the microhooks.

The muscle-mimicking phantom assumed a muscle tissue T1 also of 1400 ms, but a lower T2 of 35–40 ms at 3T.^13^ The muscle phantom sample was similarly prepared but with 3.5% agarose and no gadolinium. Once the agarose had dissolved, half of the mixture was poured into the glass jar and left to cool to room temperature. The nerve-mimicking cylinders wrapped in NT were placed flat and parallel to one another on the muscle-mimicking phantom (Fig. 1A). The remaining hot, muscle-mimicking mixture was then poured into the glass jar and allowed to cool and solidify. Silicon sealant was applied to the glass lid to minimize evaporation.

### MRI and Ultrasound Technique

MRI was performed on a 3T system (Signa Premier; GE Healthcare, Waukesha, Wis.). For phantom imaging, the glass jar was positioned parallel to the ground within an 18-channel transmit/receive knee coil for acquiring different scan planes – coronal (longitudinal, or top view) and axial (cross-sectional view). The pulse sequence parameters used are listed in Table 1.

**Table 1.** MRI Parameters

Parameter/Sequence	2D Proton Density Fast Spin Echo		3D Zero Time to Echo
Orientation	Coronal	Axial	Coronal
Field of view, mm	140	120	140
Matrix size	512 × 352	512 × 352	172 × 172
Flip angle, degrees	90/140	90/140	1
Slice thickness, mm	1	1	0.8
Repetition time, ms	4500	4500	564
Echo time, ms	24	24	0.02
Bandwidth, Hz/pixel	325.5	352.5	488.3
Echo train length	16	16	-
Acquisition time (min:s)	02:33	04:21	04:27

To measure the length of each NT, a research assistant (M.A.) utilized Volume Viewer (GE Healthcare), whereby the distance of the signal voids deemed to be produced by the microhooks were measured on the coronal image that best showed the entire length of the nitinol columns. The same software was then used to obtain the diameter of the cylinders using the microhooks to help visualize the internal diameter of the cylinders. This method was chosen, as the diameter of each NT itself could not be measured directly due to inadequate visualization of the SIS backing of the NT. To obtain the microhook (signal void) height, a single-axial image plane was used, whereby the radial distance of each signal void from the SIS backing to the tip of the microhook was measured across all visible signal voids (eight signal voids for the 7-mm devices and six signal voids for the 4-mm device) to obtain mean and SD values.

High-resolution ultrasound was performed using either a 4- to 18-MHz linear transducer or 3- to 22-MHz “Hockey-Stick” transducer on a Samsung RS85 scanner (Samsung NeuroLogica, Danvers, Mass.) by a musculoskeletal radiologist (Y.E.) with 13 years’ experience in peripheral nerve ultrasound.

### Cadaver Limb Model Imaging Investigation

A cadaveric limb investigation was performed on one left forearm to evaluate subsequent imaging visualization of NT and to assess the accuracy of NT lengths and locations on imaging (relative to those measured at the time of implantation). Two plastic surgeons trained in peripheral nerve and microsurgery (L.G. and A.B.V.) transected the nerves and implanted all NT devices at a total of four locations in a single cadaveric limb: median nerve (proximal forearm), median nerve (distal forearm), index finger, palmar ulnar digital nerve, and the ulnar nerve just distal to the elbow (not analyzed in this work). Each nerve was transected at two sites: approximately 3 cm apart for the median nerve and 1.5 cm apart for the digital nerve, whereby NT was placed at one transection site and the other was repaired using standard 9-0 nylon microsutures. In the distal forearm and index finger, the proximal site was repaired with microsuturing, whereas the distal site was repaired with NT. In the proximal forearm, the distal site was repaired with microsuturing, whereas the proximal site was repaired with NT.

MRI was performed approximately 1 hour postsurgery, using the techniques described for the phantom imaging. Ultrasound was performed approximately 4 hours postsurgery, by the same radiologist who conducted the ultrasound on the phantom.

For analysis, visualization of MRI features and distance measurements were performed by a musculoskeletal radiologist (D.B.S.) with 9 years’ experience. Focal nerve discontinuity was deemed to be the site of the microsuture repair.

## RESULTS

### Phantom Imaging

MRI of the phantom, acquired longitudinally and axially with respect to the nerve components, demonstrated signal voids that corresponded to the NT microhooks, without appreciable distortion to the cylindrical shape of the nerve-mimicking components. The nerve components demonstrated mild hyperintensity relative to the muscle components on 2D proton density fast spin echo (Fig. 1). Ultrasound of the phantom demonstrated hyperechoic foci corresponding to the microhook positions and the interface of the SIS material of the NT between the nerve- and muscle-mimicking phantom components (Fig. 1).

The dimensions of the three nerve repair devices, assessed on both longitudinal and axial MR images, closely aligned with the specifications provided by the manufacturer (Table 2). Deviations in length were within 1.3% for MRI and 14.0% for ultrasound, whereas deviations in diameter were within 19.3% for MRI and 10.6% for ultrasound. However, the mean height of the microhooks in these devices exceeded the design microhook height by 100% to 118% on MRI. Ultrasound measurements of microhook heights exceeded design specifications by 41.8% to 74.7%.

**Table 2.** Measurements of Three NT Devices Embedded within Muscle Phantom

		Design			MRI			Ultrasound		
	NT	NT_1_	NT_2_	NT_3_	NT_1_	NT_2_	NT_3_	NT_1_	NT_2_	NT_3_
Dimension	Length, mm	22	16	22	21.96 ± 0.06	15.79 ± 0.07	21.85 ± 0.10	21.41 ± 0.01	18.25 ± 0.15	22.77 ± 0.04
	Diameter of cylinder, mm	9	5	7	8.87 ± 0.12	4.03 ± 0.12	7.13 ± 0.17	8.94 ± 0.04	4.47 ± 0.09	7.01 ± 0.02
	Microhook height, mm	0.55	0.475	0.55	1.20 ± 0.04*	1.03 ± 0.08*	1.10 ± 0.03*	0.78 ± 0.07*	0.83 ± 0.04*	0.79 ± 0.03*

Mean NT diameter was measured on MRI and ultrasound by measuring the diameter of each nerve-mimicking cylinder three times and taking the average.

* Mean microhook heights (and SDs) were measured on MRI and ultrasound by averaging across all visible microhook columns on an axial image plane.

### Cadaveric Limb Imaging

**Fig. 2.**
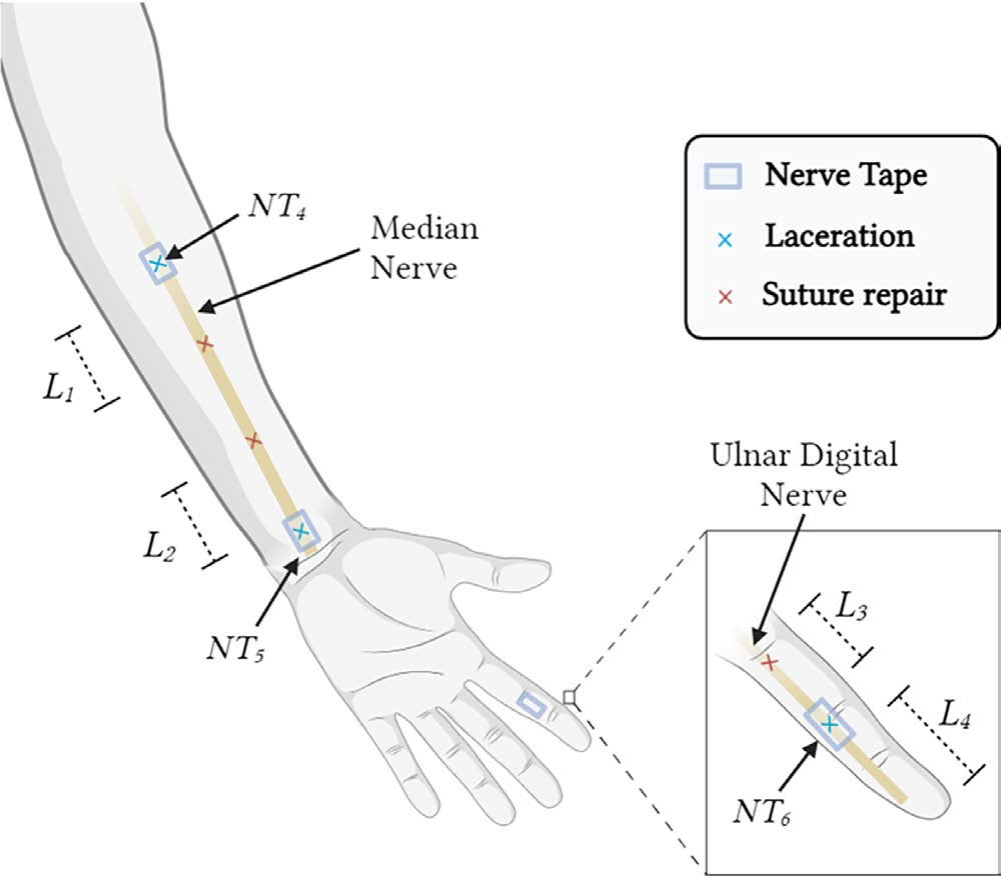
Schematic of cadaveric limb displaying the relative locations of the implanted NT devices and suture repairs. L_j_, length for 1 ≤ j ≤ 4; NT_i_, NT for 4 ≤ i ≤ 6. Created with BioRender.com.

Figure 1 illustrates locations of the nerve transections and NT repairs on the cadaveric limb, including two sites along the median nerve (NT_4_ and NT_5_) and one along the ulnar digital nerve of the index finger (NT_6_). An additional transection, with subsequent nerve suturing, was performed at each site, in proximity to each NT device (Fig. 2).

**Fig. 3.**
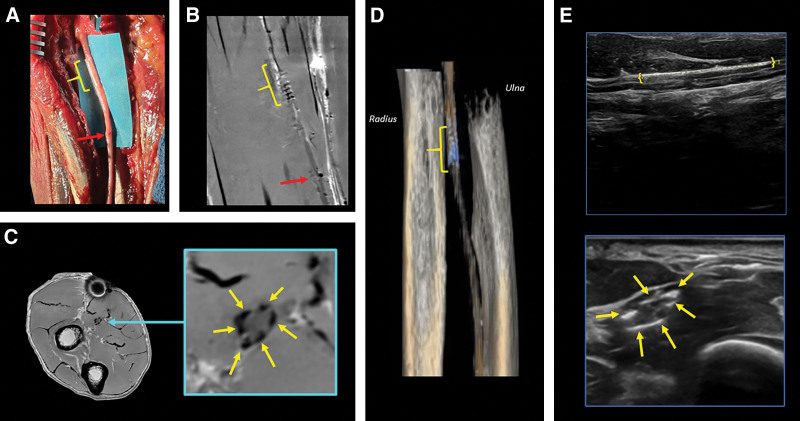
Median nerve in the cadaveric proximal forearm. A, Surgical implantation of NT (NT_4_, yellow bracket) at a transection site, and suture repair at a different transection site (red arrow). B, A coronal proton density MR image slice of the nerve longitudinally demonstrates signal void patterns from NT_4_ microhooks and the proximal nerve transection site due to the discontinuous nerve appearance (red arrow). C, Axial MRI depicts signal void patterns of the microhooks around the median nerve (yellow arrows, magnified view). D, Volume rendering of the radius, ulna, and NT_4_ (blue) around the transection site (bracket) from 3D ZTE MRI and the nerve segmentation from coronal proton density images. E, Ultrasound images. Top: longitudinal view of the median nerve and length of NT (yellow brackets). Bottom: axial view with echogenic foci demonstrating the position of the microhooks around the median nerve.

**Fig. 4.**
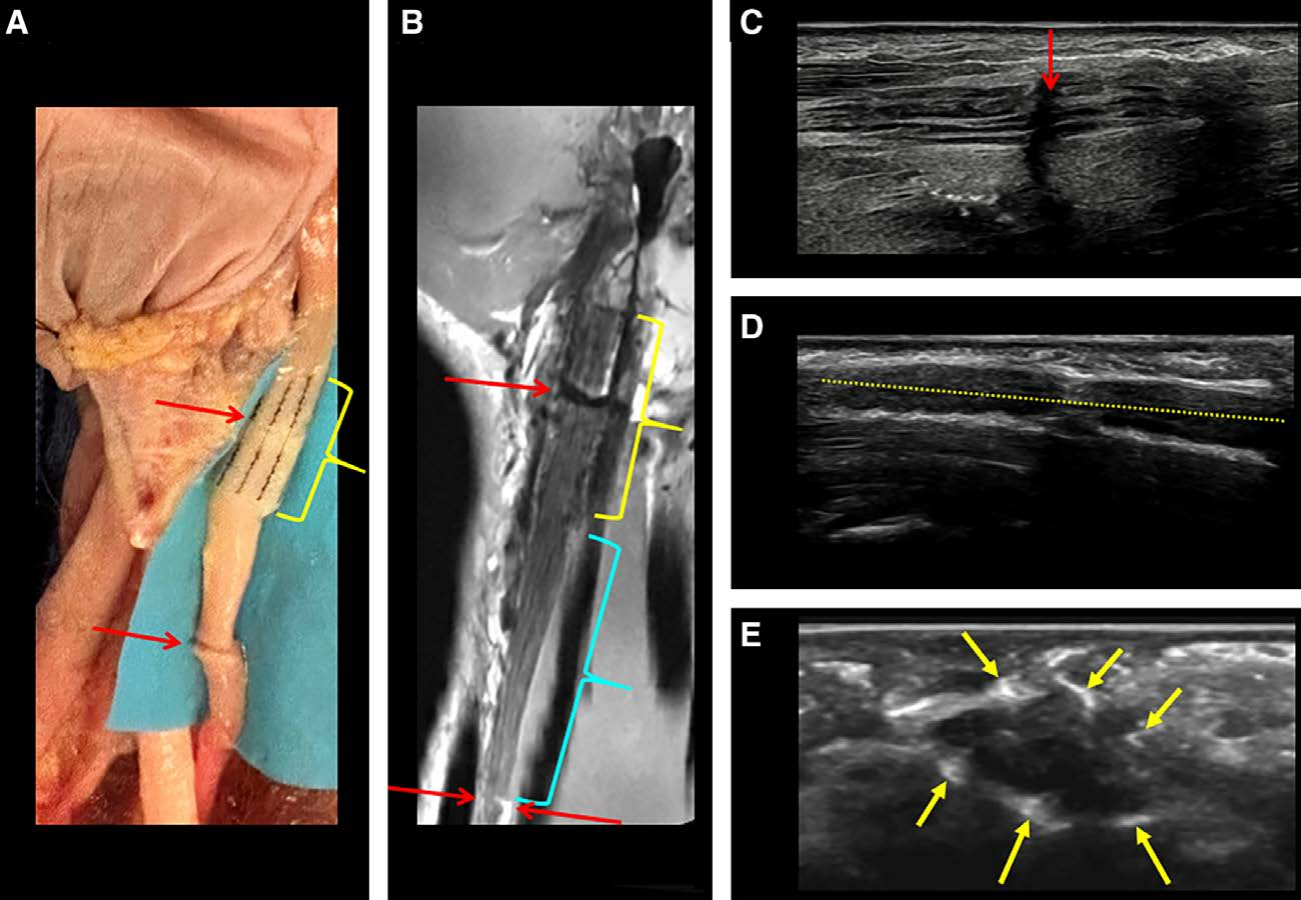
Median nerve in the cadaveric distal forearm/proximal wrist. A, Surgical implantation of NT device (NT_5_, yellow bracket) and transection sites (red arrows), with the bottom (proximal) transection repaired with sutures. B, The corresponding coronal proton density MRI demonstrates the longitudinal nerve course, NT_5_ signal void patterns (yellow bracket), and discontinuous nerve appearances at both transection sites (red arrows). The interval (L_2_) between the proximal end of NT_5_ and the proximal transections is indicated (cyan bracket). C, Longitudinal ultrasound images demonstrate both the proximal median nerve transection repaired by suture (red arrow), and (D) hyperechoic signal along the NT (yellow dotted line), whereas (E) axial ultrasound demonstrates the hyperechoic foci of the microhooks (arrows).

**Fig. 5.**
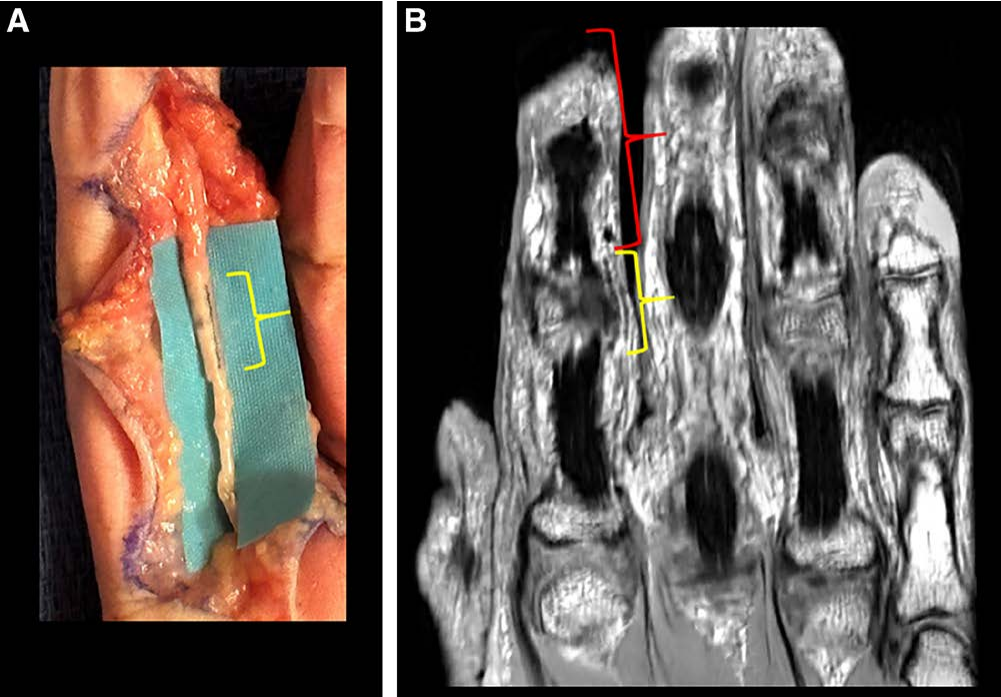
Cadaveric index finger palmar, ulnar digital. A, Surgical implantation of NT (yellow bracket, NT_6_), at the proximal interphalangeal joint level. B, Coronal proton density MR image demonstrates signal voids along NT_6_ (yellow bracket) and the approximate interval (L_4_) from the fingertip to the distal end of the device (red bracket, fingertip appreciated from a different slice, not shown).

On MRI, the implanted NT devices exhibited similar signal void patterns to those seen in the phantom, on both longitudinal and axial slices. Specifically, these were observed in both the median nerve (Figs. 3, 4) and digital nerve (Fig. 5). Nerve suture locations of the median nerve were approximated by a discontinuous nerve appearance on longitudinal images (Figs. 3, 4). The nerve suture site of the digital nerve was not well visualized, as the discontinuity from transection could not be confidently appreciated.

On ultrasound, the nerve suture itself was not visualized, but the transection site was visible only in the distal median nerve (Fig. 4). The NT placed around the median nerve displayed higher echogenicity in the regions of the microhooks, on both longitudinal and axial images (Fig. 4). The digital nerve was not well visualized on ultrasound, and therefore, no measurements were made.

Table 3 compares distances measured intraoperatively with those measured on MRI, corresponding to those illustrated in Figure 2. Distances measured by MRI closely aligned with surgical measurements, with errors within 1.7% for L_1_, L_2_, and L_4_. Table 3 also compares the manufacturer-specified lengths of NT devices against MRI and ultrasound. The errors were within 4.1% for MRI measurements of NT_4_, NT_5_, and NT_6_. For ultrasound measurements of NT devices around the median nerve (NT_4_ and NT_5_), the differences ranged from 1.9% to 3.2%.

**Table 3.** Cadaveric Limb NT Dimensions and Implantation Distance Measurements

	Measurements	Intraoperative/Device Manufacturer Specifications	MRI	Ultrasound
Median nerve (proximal forearm, Fig. 3)	L_1_ (mm)	30.0	30.5	Not measured
	NT_4_ length (mm)	16.2 ± 0.9	16.5	16.3
Median nerve (distal forearm, Fig. 4)	L_2_ (mm)	31.0	31.3	Not measured
	NT_5_ length (mm)	22.0 ± 0.9	21.1	21.3
Ulnar digital nerve (index finger, Fig. 5)	L_3_ (mm)	15.0	Not measured*	Not measured
	L_4_ (mm)	36.0	36.0	Not measured
	NT_6_ length (mm)	14.0 ± 0.9	14.5	Not measured

* Suture was not visible.

## DISCUSSION

This study demonstrates the qualitative appearance and dimensional measurements of NT, a novel peripheral nerve repair device, with its microhooks manifesting as signal voids on MRI and hyperechoic foci on ultrasound. The lengths of NT and locations observed on both MRI and ultrasound matched ground truth measurements with relatively good accuracy (within 4.1% for MRI and 3.2% for ultrasound). However, the size of the signal voids on MRI were about twice the specified height of the NT device; this disparity is attributable to the magnetic susceptibility effects from nitinol (χ = 6.6 × 10^−6^), which were well visualized in phantom and cadaveric nerve implantation images. Similarly, ultrasound also overestimated the height of the NT device, and this could be attributed to comet tail and shadowing effects.

The larger NT devices (on the median nerve) were both well-visualized on MRI and ultrasound, but the smaller device (on the digital nerve) was not as well defined on MRI and not visualized on ultrasound. This likely reflects limitations of resolution and signal-to-noise ratio afforded by the imaging modalities, which in MRI primarily depends on the field strength and choice of MRI parameters, and in ultrasound depends on transducer frequency and imaging depth. On MRI, imaging at a higher field strength may improve the achievable spatial resolution and increase the detection of susceptibility patterns of the microhooks.

Another limitation encountered was the presence of air bubbles postsurgery, which introduced susceptibility patterns (χ = 3.6 × 10^-7^) manifesting as signal voids. In a clinical scenario, air bubbles would be expected to dissipate within 14 days or might otherwise indicate possible postoperative infection^15^; the optimal duration for postimplantation imaging was not evaluated in this work. Another limitation of this work was additional susceptibility patterns from the geometry of the amputated cadaver limb, which resulted in difficulties with MRI of the fourth ulnar nerve implantation site close to the stump that was therefore not analyzed in this work.

Magnetic susceptibility effects on MRI have been previously well characterized.^16^ Magnetic susceptibility disrupts the local magnetic field, resulting in both spatial distortion (from spatially varying magnetic field) and signal voids (from radiofrequency signal outside receiver bandwidths, as resonant frequency is proportional to the magnetic field). The combination of distortion and signal voids can also cause “ringing” artifact, which was not observed in this work due to the relatively small susceptibility effect of nitinol [as compared with materials with much greater susceptibility such as martensitic stainless steel (χ = 0.4–1.1 × 10^3^) and cobalt (χ = 2.5 × 10^2^)]. Using lower magnetic field strengths, higher receiver bandwidths, higher through-plane resolution, and multispectral imaging techniques^17^ can reduce magnetic susceptibility effects. These effects also vary with the relative orientation to the main magnetic field and are most pronounced at 0 and 90 degrees. This preliminary work assessed the MRI appearance of NT but did not evaluate how these imaging parameters affected the visibility of the device itself.

Although MRI is uncommonly performed after nerve surgery,^18^ it has also been proposed for assessing axonal recovery.^19^ Postsurgical recovery is commonly assessed via physical exam and sometimes with electrodiagnostic testing. However, the time to reinnervation varies and hinges on factors such as nerve length.^20^ Nerve growth has been estimated at 1 mm per day,^21,22^ with typical motor function recovery times between 2 and 5 years^23^ and electrodiagnostic testing first detecting motor unit potentials 3–19 months after repair.^24^ Furthermore, functional recovery rates after nerve repair vary with patient age (younger patients typically display better sensory recovery),^25,26^ muscle fiber size, time from injury to repair, nerve repair site (proximal repairs are associated with better motor recovery than distal repairs), and tissue bed conditions.^23^ Therefore, postrepair peripheral nerve imaging may have prognostic value. Specifically, imaging of the coaptation site itself, and of the entire length of the nerve after repair, may provide noninvasive characterization of the integrity of the nerve repair site, providing indications of axonal regeneration. However, as mentioned above, precise location of the coaptation site can be difficult, in part due to limited MRI and ultrasound visibility of microsutures. This study suggests that repairs performed with NT may be more reliably localized for imaging, potentially facilitating assessment of repair site integrity and continued advancements toward image-based monitoring of nerve regeneration. As the results of this cadaver study are preliminary, further imaging studies of NT implantation in human subjects will be needed to confirm the imaging features in patients and determine the feasibility for assessing peripheral nerves post repair.

## DISCLOSURES

Drs. Sneag and Tan receive institutional research support from GE Healthcare, Siemens Healthcare, and AMAG Pharmaceuticals. Dr. Clements is an employee at BioCircuit Technologies, Inc. The other authors have no financial interest to declare in relation to the content of this article. This study was funded in part by NIH National Institute of Neurological Disorders and Stroke grant SB1NS137879.

## Supplementary Material


