# Supplementary figures and images for: MRI and Ultrasound Visualization of a Nerve Repair Implant Containing Nitinol
Source: Plast Reconstr Surg Glob Open. 2024 Aug 9;12(8):e6063. doi: 10.1097/GOX.0000000000006063 (PMC11315573; doi:10.1097/GOX.0000000000006063)

(A)

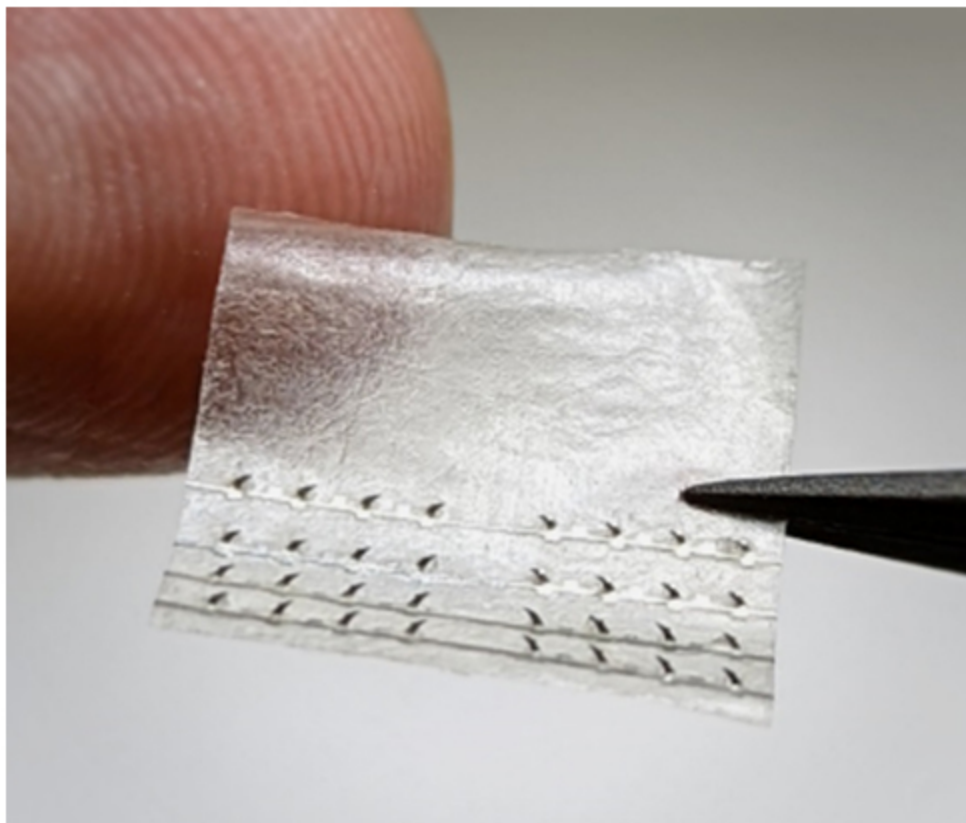

(B)

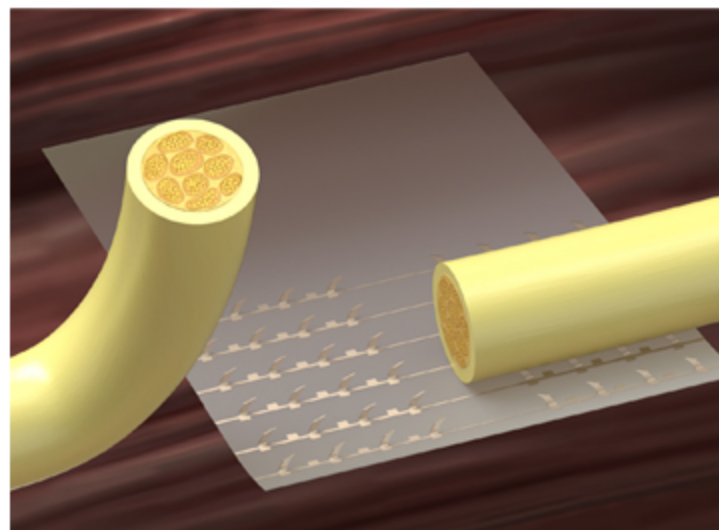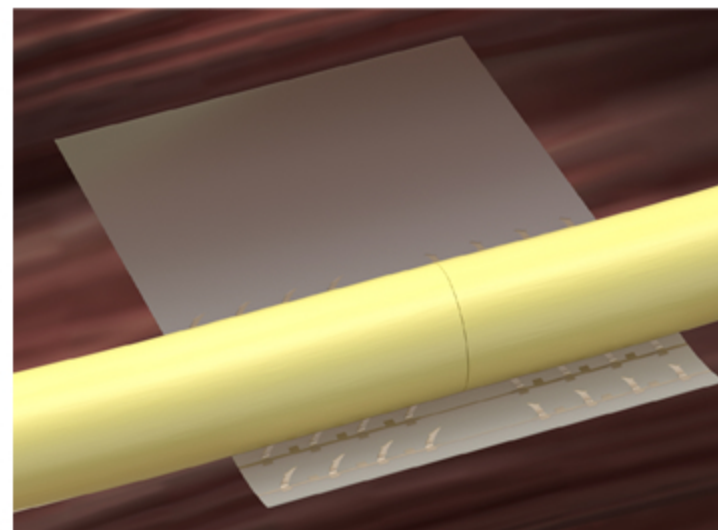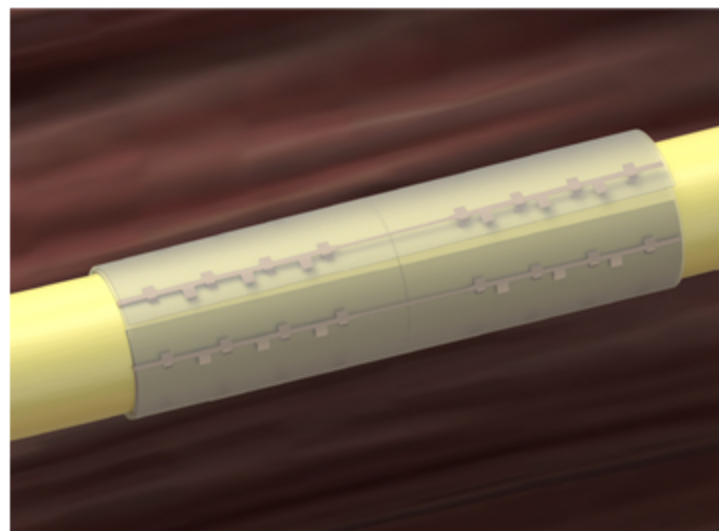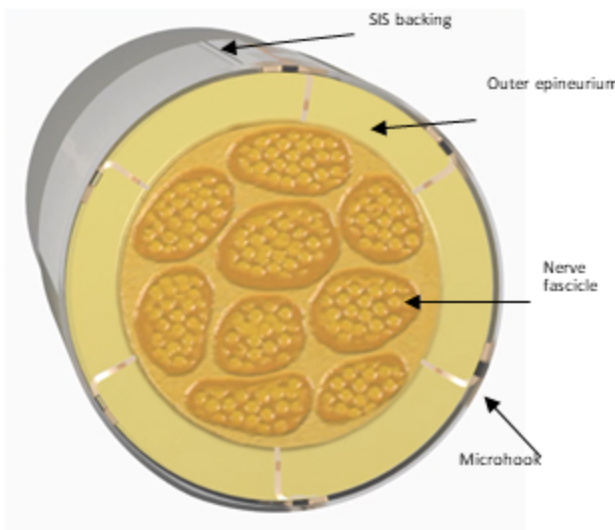

Supplement: Supplementary file 1 [file gox-12-e6063-s001.pdf]
